# Recent advances in understanding tumor stroma-mediated chemoresistance in breast cancer

**DOI:** 10.1186/s12943-019-0960-z

**Published:** 2019-03-30

**Authors:** Jana Plava, Marina Cihova, Monika Burikova, Miroslava Matuskova, Lucia Kucerova, Svetlana Miklikova

**Affiliations:** Cancer Research Institute, Biomedical Research Center, Slovak Academy of Sciences, Dúbravská cesta 9, 845 05 Bratislava, Slovakia

**Keywords:** Breast cancer, Chemo-resistance, Molecular mechanisms, Tumor stroma, Mesenchymal stromal cells

## Abstract

Although solid tumors comprise malignant cells, they also contain many different non-malignant cell types in their micro-environment. The cellular components of the tumor stroma consist of immune and endothelial cells combined with a heterogeneous population of stromal cells which include cancer-associated fibroblasts. The bi-directional interactions between tumor and stromal cells therefore substantially affect tumor cell biology.

Herein, we discuss current available information on these interactions in breast cancer chemo-resistance. It is acknowledged that stromal cells extrinsically alter tumor cell drug responses with profound consequences for therapy efficiency, and it is therefore essential to understand the molecular mechanisms which contribute to these substantial alterations because they provide potential targets for improved cancer therapy. Although breast cancer patient survival has improved over the last decades, chemo-resistance still remains a significant obstacle to successful treatment.

Appreciating the important experimental evidence of mesenchymal stromal cells and cancer-associated fibroblast involvement in breast cancer clinical practice can therefore have important therapeutic implications.

## Introduction

Breast cancer is the most frequent cancer diagnosed in women, and is one of the greatest causes of global female death. In addition, the American Cancer Society reports this is 25% of all new cancer diagnoses in women world-wide (American Cancer Society, Cancer Facts and Figs. 2017). Breast cancer is a heterogeneous disease classified in the following three main groups based on immuno-histochemical analysis: (I) estrogen receptor ER(α)-positive, (II) human epidermal growth factor receptor Her2 positive and (III) triple negative (ER(α)-negative, progesterone receptor (PR) negative and Her2-negative. Further sub-typing is based on gene expression profiling which unraveled the gene cluster which is mostly expressed in luminal breast cells, myo-epithelial basal cells and cells associated with increased expression of Her2.

These sub-types are named “luminal-like, basal-like and Her2-enriched” [1], and the profiling also identified clinically important sub-types in these three molecular groups. For example, the luminal A and B subtypes induce different patient prognosis, where patients carrying the luminal B tumor type have worse prognosis [2, 3] and the basal-like and claudin^low^ subtype express mesenchymal markers such as vimentin. While this is present in epithelial tumors, it is not a component of normal breast tissue [4].

However, tumor cells alone do not drive tumor growth or progression. Despite early detection and increased knowledge of breast cancer biology, approximately 30% of patients with breast cancer experience recurrence. The relapse usually occurs in patients with adenocarcinoma cells with chemo-resistant phenotype; and while this was previously linked to tumor cell genetic alterations, it is now acknowledged that adjacent tissue surrounding tumor cells has an important role in tumor progression and resistance [5]. It is also evident that many “normal” cells add to tumor diversity by varying the micro-environment composition, stromal cell proportions and/or activation states.

In addition to malignant cells and various non-malignant cell populations, solid tumors also contain an extracellular matrix (ECM) which forms a complex tumor micro-environment (TME) or tumor stroma. These stromal cells, ECM, soluble factors and the physical state of the tumor microenvironment all affect solid tumor behavior in a complex manner [6]. Moreover, TME is now considered a hallmark of cancer biology [7], and researching the molecular characteristics and interactions between TME components and tumor cells is expected to produce important knowledge for developing new therapeutic approaches.

Tumor drug responses are not exclusively determined by the tumor cell’s intrinsic characteristics because tumor-associated stromal cells, including fibroblasts, mesenchymal stromal cells (MSCs), immuno-inflammatory cells, vascular endothelial cells and the ECM combine in response to anti-cancer treatment. These components influence the proliferation, invasion and metastasis of tumor cells [8], and the adjacent adipose tissue provides a rich source of MSCs which significantly contribute to stromal constituents in the breast cancer tumor micro-environment.

Many experimental studies have also confirmed that MSCs interact with breast cancer cells. They possess “homing ability” to breast cancer tissue and release growth factors which consequently promote migration and epithelial-to-mesenchymal transition (EMT). However, different reports on MSCs’ influence on chemotherapy response have produced contradictory findings, and while some studies have reported that MSCs contribute to increased breast cancer cell chemo-resistance [9–12], our results indicate that MSCs may even act as a drug sensitizer [13, 14].

To improve insight into tumor development and chemotherapeutic approaches, it is most important to understand the interplay between specific TME components, the associated cellular communication processes and resultant interactions of this network between cancer cells and the various tumor-associated cell populations. Here, we focus on the molecular communication between stromal cells, mainly MSCs and breast cancer cells, and the cell-to-cell signaling role and its effect on chemotherapy efficiency.

### Cellular components of stroma in breast tumors

Tumor tissue is a heterogeneous mixture of cells, where cancer cells are surrounded by disorganized blood vessels formed by endothelial cells, lymphatic vessels, infiltrated immune cells (T cells, natural killers (NKs) and macrophages), adipocytes, fibroblasts and MSCs. Some of these cells exist in the tissue before tumor development and others are recruited to the micro-environment by the tumor cells [15, 16]. TME heterogeneity depends on location within the tumor, and TME cells located at the tumor periphery can significantly differ from cell types at the tumor core [17]. This is due to randomly generated mutations in the tumor cells, immune cell infiltration, tumor cell necrosis and interstitial pressure [18]. While each tumor has unique TME, critical TME components and their role in tumor progression remain similar in different cancers. Bi-directional communication between cells and their micro-environment is necessary for normal tissue homeostasis. However, it is also required for tumor growth, and therefore interaction between cancer cells and the surrounding stroma is an important relationship that alters all cell phenotypes, proliferation and metabolism. This communication also affects disease initiation and progression; and thus influences patient prognosis [19, 20].

This review specifically focuses on novel findings in the contribution of MSCs and cancer-associated fibroblasts (CAFs) in breast cancer chemo-resistance. Although the link between MSCs and CAFs remains undetermined, recent studies suggest they may have similar characteristics and pro-tumorigenic activity. In contrast, however, Su et al. found no overlap between these components [21, 22].

### Mesenchymal stromal cells

Mesenchymal stem/stromal cells are multipotent spindle-shaped cells first described in the 1960’s as hematopoietic bone marrow supportive cells [23, 24]. Multiple populations of MSCs have now been derived from the plethora of adult and fetal tissues reviewed by Ullah and colleagues [25].

The term “mesenchymal stem cells” was popularized by Arnold Caplan many years later, in the belief that they can give rise to bone, cartilage, tendon, ligament, marrow stroma, adipocytes, dermis, muscle and connective tissue [26]. The International Society for Cellular Therapy (ISCT) recommended the term “multipotent mesenchymal stromal cells” because support for their “stemness” in vivo was lacking [27] and it further proposed minimum criteria to define MSCs [28]. The expression of negative surface marker CD34, however, remains controversial [29].

These characteristics are valid for all MSCs, but some differences still exist in isolates derived from various tissue types. Many studies reported additional MSC markers dependent on aspiration source. For example, stromal precursor antigen-1 (Stro-1) was identified as a “stemness” marker for the MSCs [30] and dental [31] and bone marrow-derived MSCs (BM-MSCs) [32] were reported to be Stro-1 positive while the adipose tissue-derived (AT-MSCs) are negative [33].

BM-MSCs and AT-MSCs share many important characteristics and few differences [34]. The AT-MSCs are more genetically stable in long-term culture, have lower senescence ratio, higher proliferative capacity and retain their differentiation potential for a longer period in culture than BM-MSCs [35]. In addition, AT-MSCs support haematopoiesis both in vitro and in vivo more efficiently than the BM-MSCs [36], and they also have significantly higher angiogenic potential [37]. Moreover, higher numbers of AT-MSCs are easily isolated from subcutaneous adipose tissue aspirate. This operation can be repetitive utilizing liposuction with minimal invasiveness, thus rendering this an attractive MSC source [38].

Defined by their ability to differentiate into multiple stromal cell lineages, the MSCs can be found in most body parts and they can migrate throughout the organism and into tumor tissue [39]. Therefore, tumors are sometimes considered “wounds that do not heal” because of chronic inflammation, immune cell infiltration and neo-vascularization [40]. Migration of MSCs to injury enables TME to recruit these cells by releasing inflammatory molecules, growth factors and cytokines. Although they preferentially “home and engraft” in tumors from the bone marrow, which is the major MSC reservoir, they also emanate from surrounding adipose tissue.

In addition, MSCs in TME can easily differentiate into CAFs [41], and MSC-like CAFs that express FSP and FAP [42] originate from BM-MSCs whereas the AT-MSCs mainly differentiate into vascular and fibro-vascular stromal cells [43, 44]. Here, it is also important to note that normal healthy tissues have almost no detectable FAP expression.

MSC migration to tumors leads to cellular interactions with tumor cells and TME components. This occurs both directly through gap junctions, membrane receptors and nanotubes and indirectly by soluble molecules [45]. The MSCs stimulate adjacent cells by releasing endocrine and paracrine signals. In turn, MSCs can be stimulated by tumor cells and develop an aberrant tumor-associated phenotype [46]. Consequently, they either promote or inhibit tumor cell growth [47, 48].

Reduction of tumor growth by MSCs can be mediated by inhibiting angiogenesis, suppressing the Wnt and AKT signaling pathways or inducing cell cycle arrest and apoptosis [46, 47, 49]. Thus, aberrant tumor-associated MSCs can acquire different functions after interaction with tumor cells. These include TGF-β secretion which contributes to both EMT and immune system suppression. Moreover, these MSCs release VEGF for neo-vascularization in the TME and produce CXCL12 to support tumor cell growth and survival [50]. While P2X signaling was recently identified as a pathway favoring MSCs-mediated breast cancer cell proliferation [51], high IFN-β expression suppresses human breast cancer cell growth [52]. Therefore, TME MSCs have either pro- or anti-tumorigenic properties depending on the cancer cell properties and the experimental settings [53].

Bartosh et al’s seminal research identified the remarkable phenomenon of cancer cell cannibalism and the acquired senescence-associated secretory phenotype (SASP). The authors discovered that breast cancer cells in 3D co-cultures entered dormancy after internalizing and degrading human BM-MSCs. The cannibalistic breast cancer cells then became highly resistant to chemotherapy and other stresses caused by nutrition deprivation. Most interestingly, these secreted SASP factors enabled dormant breast cancer cells to communicate with the various TME components [54].

MSCs provide a promising tool for many types of anti-tumor therapies because of their role in the TME; and this was comprehensively summarized in Valkenburg et al [55].

### Fibroblasts in breast cancer

Fibroblasts are non-vascular, non-inflammatory, non-epithelial cells in connective tissue. They secrete the extracellular matrix (ECM) and basement membrane components, regulate epithelial cell differentiation, modulate immune system responses and maintain homeostasis [56]. Activated fibroblasts are termed “cancer-associated fibroblasts” (CAFs), and they are major stromal cells contributing to the TME. When activated by direct contact with leukocytes or secreted factors, including TGF-β, PDGF, FGF2, EGF and CXCL12 [57], CAFs promote tumor growth, increase angiogenesis, degrade ECM to release signaling molecules and promote EMT and metastasis [56]. Although CAFs were first considered tumor developmental elements lacking effect on cancer cells, they have since been identified as essential components of tumor progression [58].

The CAFs can be derived not only from normal fibroblasts, but also from other types of cells, including MSCs, epithelial cells, pericytes, adipocytes and endothelial cells [59]. An interaction between tumor-induced fibroblast activation and fibroblast-induced tumor proliferation and metastasis has been proven, thus it can be concluded that CAFs act as tumor supporters [60].

CAFs are present in the TME in aberrantly high numbers and differ from normal fibroblasts in many morphological and biological ways. CAFs are functionally defined by intensive proliferation and high ECM deposition, and further acknowledged as “activated myofibroblasts that cannot regress to an inactivated state” [61].

The CAFs exhibit differential gene expression of several factors compared to normal fibroblasts. Membrane protein FAPα, selectively expressed in activated CAFs, is one of the most important markers of these cells [62], and FSP-1, podoplanin-a, S100A4 protein, vimentin and PDGF receptors α and β are also highly expressed in CAFs [63]. Most recently, the IGFBP7 protein has been identified as a novel biomarker of tumor fibroblasts. IGFBP7-expressing CAFs have been demonstrated to promote colon cancer cell proliferation through paracrine tumor-stroma interactions in vitro [64]. In addition, TGF-β2 expression in CAFs was previously identified in metastatic colon cancer [65].

In summary, 46 differentially expressed genes regulated by the transforming growth factor (TGF)-β signaling pathway were identified in CAF cell lines compared to normal fibroblast cell lines [66]. All these genes encode for paracrine factors released into the TME. Moreover, numerous altered gene transcripts have been identified in breast CAFs, including ribosomal protein S6 kinase α3, FGF receptor 1, nardilysin and cyclin-dependent kinase inhibitor 1B [67].

Su et al. also recently identified the CD10 and GPR77 fibroblast-associated cell-surface molecules not previously described. These specifically define a CAF sub-population which promotes chemo-resistance and cancer formation in breast and lung cancer patients. CD10^+^GPR77^+^ CAFs secrete abundant interleukins IL-6 and IL-8 which provide a survival niche for cancer stem cells (CSCs) via continuous NF-κB signaling. Although most CAFs are relatively genetically stable, and therefore present a potential therapeutic target with lower risk of developing chemo-resistance [68], increasing data suggests that fibroblasts’ protective role enables cancer cells to evade the cytotoxic effects of chemotherapy. For example, HGF has been identified as an essential factor in the CAF-mediated resistance to lapatinib in HER2+ breast cancer [69]; and CAFs may also act as a physical barrier against anti-tumor drugs and decrease their availability to tumor cells.

### Chemo-resistance in breast cancer and association with “stemness” phenotype

**Fig. 1:**
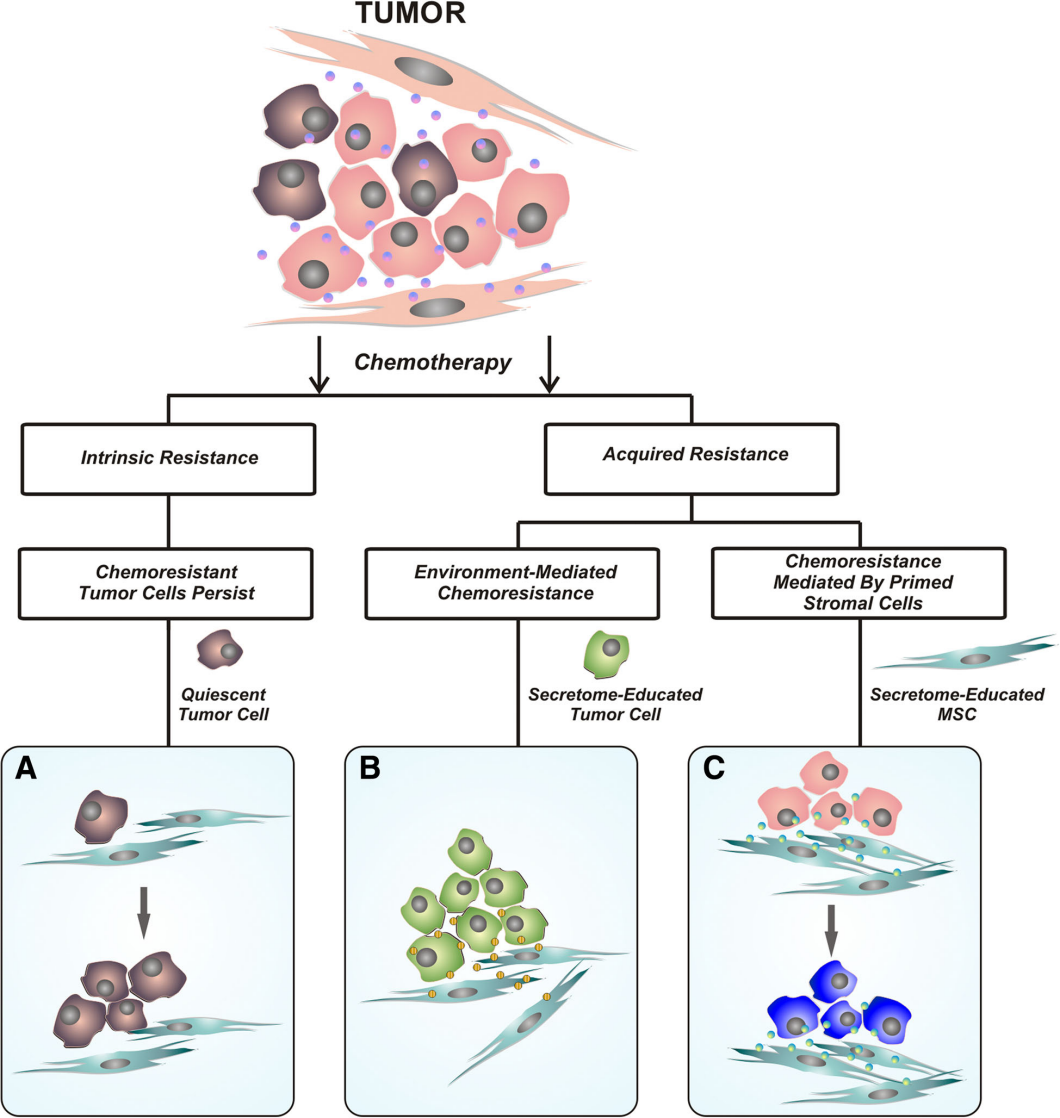
Scheme of possible outcomes following chemotherapy treatment. There are three possible outcomes from chemotherapy treatment. **a** Intrinsic resistance to chemotherapy associated with the tumor cell quiescent state is usually defined by over-expression of ABC drug transporters, different anti-apoptotic genes and a more effective DNA repair system. **b** Acquired resistance that occurs despite initial positive response to therapy; here, different signaling pathways between secretome-educated cancer cells and MSCs are involved and **c**) Acquired chemo-resistance mediated by secretome-educated stromal cells through altered secretion profiles and different signaling pathways

Chemo-resistance can be an intrinsic and inherent feature of tumor cells, where this is often associated with their quiescent state prior to treatment. In contrast, acquired resistance occurs despite initial positive response to therapy [70]. There are many mechanisms of resistance that include different involved cells and signaling pathways; dependent on the cancer type (Fig. 1). Up-regulation of the cancer stem cell phenotype can be critical in resistance to a variety of drugs in cancer treatments; including breast cancer treatment [71]. CSCs have increased capacity to actively export many drugs from cells by over-expressing ATP-binding cassette (ABC) drug transporter proteins. Moreover, CSCs have higher anti-apoptotic gene expression and a more effective DNA repair system [72].

The TME provides shelter for CSCs; thus inducing therapy resistance and tumor development. However, traditional cancer treatments, including the majority of chemotherapeutic agents and radiation, target actively dividing cells, and while they reduce tumor mass, they do not effectively remove quiescent cells such as CSCs. This can lead to tumor recurrence. For example, breast CSCs have paclitaxel resistance [73], and also decreased ROS expression which is critical in inducing DNA damage by ionizing radiation [74], but the TME blocks drug penetration and suppresses immune system responses [75]. Herein, we focus on the mechanisms involved in acquired chemo-resistance mediated by stromal cells in breast tumor TME. This is tightly linked to their mutual interactions and the “stemness-phenotype-support” exerted by MSCs and CAFs.

### Chemo-resistance mediated by mesenchymal stromal cells

MSCs usually interact with breast CSCs through IL-6 and CXCL7 cytokine secretion. This signaling is responsible for the self-renewal potential of breast CSCs. Cytokines such as SDF-1 (CXCL12) produced by CAFs can also promote the proliferation of cancer cells carrying the SDF-1 receptor CXCR4; where SDF-1 expression-level correlates with breast cancer survival [76].

**Fig. 2:**
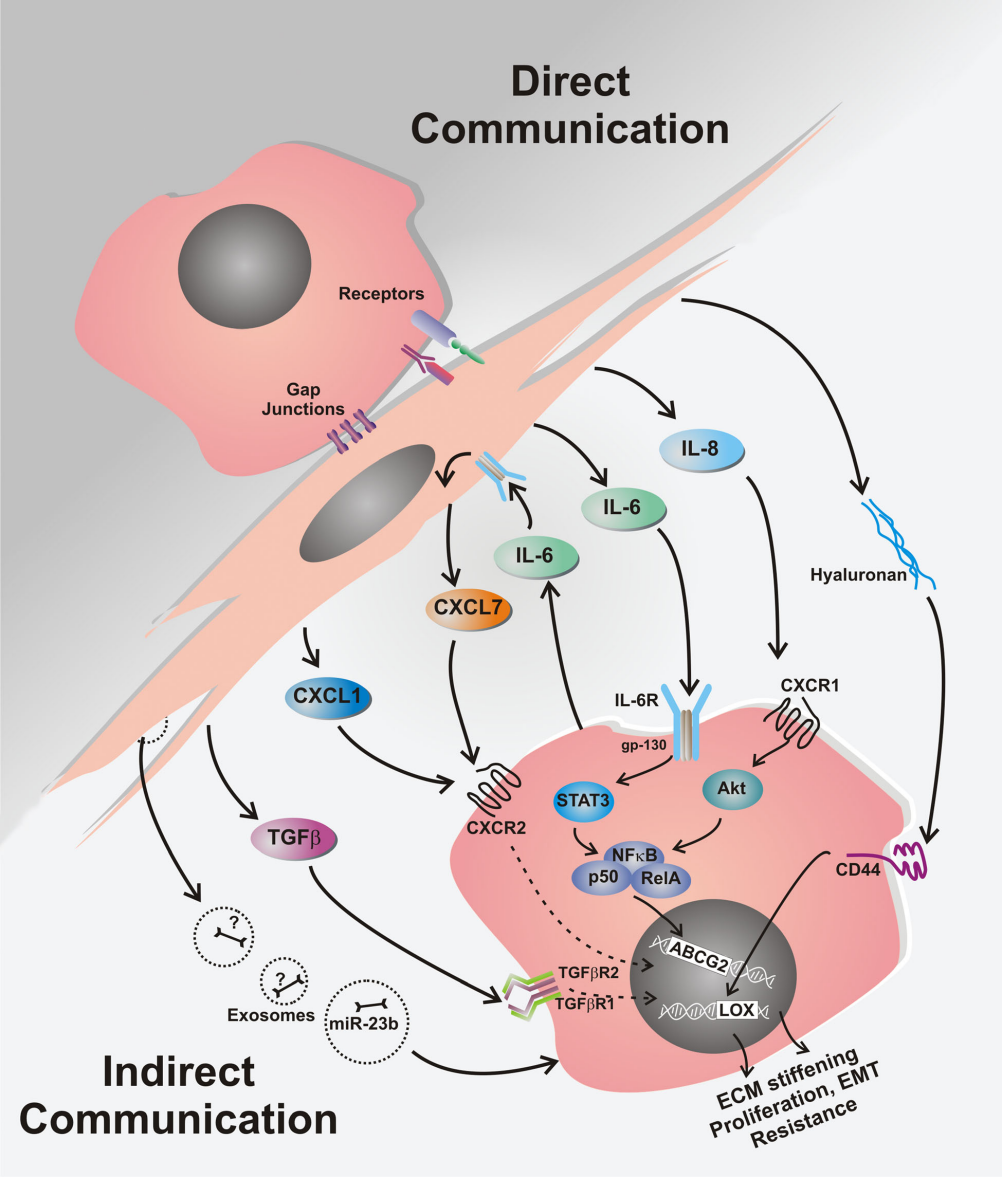
Chemo-resistance mechanisms mediated by MSCs in breast cancer. Communication between MSCs and breast cancer cells leading to resistance against cytotoxic drugs mostly involves secretory proteins. Following chemotherapy treatment, MSCs secrete increased levels of cytokines (IL-6, IL-8, CXCL1, CXCL7, TGFβ), PUFAs (not shown), plus hyaluronan, exososomes and other molecules. All these are involved in complex inter-cellular communication that ultimately manifests as a chemo-resistant cancer cell phenotype. Direct cell-to-cell interactions are also involved, especially through the functional gap junctions and connexin-interacting proteins and direct receptor communication

Multiple signaling pathways have been ascribed to the MSC- and CAF-mediated drug resistance in breast cancer, and these are often associated with induction of the “stemness” phenotype. Moreover, the protective effect of MSCs on breast cancer cells against cytotoxic drugs appears to need both secretory proteins and direct cell-to-cell interaction (Fig. 2). Here, IL-6 has an important role in acquired breast cancer chemo-resistance through its secretion by MSC which promotes great impact on the stimulation of ERα-positive breast cancer cell proliferation [77, 78]. In addition, IL-6 has proven protective effect against paclitaxel and doxorubicin in ERα-positive breast cancer [9, 79], and also against trastuzumab in Her-2 positive tumors [80]. However, IL-6 released by breast cancer cells mediates “homing” of MSCs into primary tumor sites, and then interacts with its MSC receptor to induce MSC CXCL7 secretion. These cytokines work together to provide chemokine networks that influence CSCs to promote resistance to anti-cancer drugs [81].

The MSC secreted CXCL1 cytokine enhances expression of ABCG2 by altered miR-106a expression in triple negative breast cancer cells. ABCG2 is also known as the breast cancer resistance protein (BRCP) and it is the ATP-binding cassette transporter protein responsible for the efflux of doxorubicin, and causes resistance to this drug [11]. Similarly, IL-8 secreted by MSCs increases ABCG2 expression. This results in reduced intracellular doxorubicin accumulation in triple negative breast cancer cells [10].

MSCs also produce abundant levels of transforming growth factor β (TGF-β) and this signaling pathway can trigger epithelial to mesenchymal transition (EMT). Thus, TGF-β contributes to the MSC drug protective effect by inducing EMT. MSCs can also promote EMT by the different mechanism of producing hyaluronan which enables MSCs to make CD44-expressing breast cancer cells produce lysyl oxidase (LOX). This then causes ECM stiffening by catalyzing collagen fiber cross-linking, and facilitates ECM-induced drug resistance [82]. Moreover, it also stimulates the expression of the Twist EMT-mediating transcription factor. In addition, hyaluronan can also be produced by breast cancer cells as a result of MSC secretion of basic fibroblast growth factor [83, 84]. Thus, the MSCs can foster hyaluronan accumulation; and hyaluronan excess in breast tumor stroma induces doxorubicin resistance [83].

The importance of cell-to-cell interaction between MSCs and breast cancer cells in acquired drug resistance is highlighted by MSC presence inducing over-expression of HER-2 and loss of PTEN. This indicates that MSCs regulate HER-2 receptor and PTEN tumor suppressor interaction in breast cancer cells by activating Src which induces subsequent PTEN inactivation. Therefore, Src and its downstream PI3K/Akt signaling pathway enhance resistance to trastuzumab [85].

Further recent study has shown that MSCs induce increased expression of PAG1/Cbp; a transmembrane adaptor protein which enhances resistance to adriamycin hydrochloride (ADMh) [86]. PAG1/Cbp is ubiquitously expressed in lipid rafts and is significantly involved in many signaling pathways that include Src-associated signaling and the AKT/mTOR pathway. Activation of Cbp leads to activation of Src and thus it can enhance resistance to ADMh; and most likely also to trastuzumab.

MSCs also secrete factors which protect carcinoma cells against platinum-based chemotherapeutics [87]. These include two types of polyunsaturated fatty acids (PUFAs), 12-oxo-5,8,10-heptadecatrienoic acid (KHT) and hexadeca-4,7,10,13-tetraenoic acid (16:4(n-3)). In minute quantities, these both induce resistance to a broad spectrum of chemotherapeutic agents. Central enzyme blocking involved in the production of these PUFAs (cyclooxygenase-1 and thromboxane synthase) prevents MSC-induced resistance. These combined findings show that MSCs are potent mediators of resistance to chemotherapy and important targets in enhancing patient treatment efficacy [12].

In addition to the above-stated molecular mechanisms, drug resistance is also mediated by exosomes derived from MSCs. These small cell-derived vesicles contain miR-23b; an miRNA inhibiting myristoylated alanine-rich C-kinase substrate (MARCKS). This is a prominent cellular substrate for protein kinase C, and its inhibition leads to breast CSC dormancy in the metastatic niche, and thereby to docetaxel treatment resistance [88].

However, there is also evidence that MSCs can not always protect tumor cells against cytotoxic drugs. Their protective activity depends on the type of the drug and also on the type of cancer cell. In addition, some reports suggest that MSCs can act as drug sensitizers. For example, BM-MSCs can sensitize breast cancer cell lines to kinase inhibitors [89], and AT-MSCs are able to render Her-2 positive breast cancer cells more sensitive to doxorubicin and 5-fluorouracil [13]. Interestingly, while some cancer cell lines react to MSCs by cell cycle arrest, others display higher proliferation activity in their presence [8, 90, 91]. Hence, cell cycle arrest is a potent mechanism enabling cancer cells to escape cytotoxic drug effects.

## Conclusions

Future cancer therapy success depends on thoroughly understanding the many complex mechanisms involved, and establishing the pathways prominent in resistance to anti-cancer treatment. Developing methods of targeting them is then essential. There is also rapidly increasing research on the tumor micro-environment (TME) and its role in chemo-resistance acquisition, subsequent treatment failure and cancer recurrence. It is therefore critical that the TME is acknowledged as an important cancer-target strategy, and that further TME investigation is initiated.

While the TME in all breast cancer sub-types acts through a network of secreted molecules, adipose tissue is most important in mediating communication between the TME and breast cancer cells because especially in the breast it forms a major part of the tumor environment. Therefore mesenchymal stromal cells from adjacent adipose tissue, and especially the cancer-associated fibroblasts in the tumor-microenvironment, are of utmost importance in processes associated with cancer progression and resistance to therapy.

Finally, recent research stresses that stromal-cell-mediated protection against cytotoxic drugs requires both secretory proteins and direct cell-cell interactions. Therefore, further research into these processes is anticipated to provide better understanding of their effects on therapy resistance and hasten the design of effective therapeutic strategies and personalized regimens for breast-cancer patients.

## Abbreviations

ABCG2: ATP-binding cassette super-family G member 2
ADMh: Adriamycin hydrochloride
AT-MSCs: Adipose tissue-derived mesenchymal stromal cells
bFGF: Basic fibroblast growth factor
BM-MSCs: Bone marrow-derived mesenchymal stromal cells
BRCP: Breast cancer resistance protein
CAFs: Cancer-associated fibroblasts
CSCs: Cancer stem cells
CXCL: C-X-C motif chemokine ligand
CXCR4: C-X-C chemokine receptor type 4
ECM: Extracellular matrix
EGF: Epidermal growth factor
EMT: Epithelial-to-mesenchymal transition
ER: Estrogen receptor
FAPα: Fibroblast activation protein-α
FGF: Fibroblastic growth factor
FSP-1: Fibroblast specific protein 1
GPR77: G protein-coupled receptor 77
HER2: Human epidermal growth factor receptor 2
HGF: Hepatocyte growth factor
IFN-β: Interferon beta
IGFBP7: Insulin-like growth factor-binding protein 7
IL: Interleukin
ISCT: International Society for Cellular Therapy
LOX: lysyl oxidase
MARCKS: Myristoylated alanine-rich C-kinase substrate
MSCs: Mesenchymal stromal cells
NF-κB: Nuclear factor kappa- B
NKs: Natural killers
PAG1/Cbp: C-terminal Src kinase (Csk)-binding protein (Cbp) encoded by PAG1
PDGF: Platelet-derived growth factor
PR: Progesterone receptor
PTEN: Phosphatase and tensin homolog
PUFAs: Polyunsaturated fatty acids
SASP: Senescence-associated secretory phenotype
SDF-1: Stromal cell-derived factor 1
Stro-1: Stromal precursor antigen-1
TGF-β: Transforming growth factor β
TME: Tumor microenvironment
VEGF: Vascular endothelial growth factor
